# Anterior tibial displacement on preoperative stress radiography of ACL-injured knee depending on knee flexion angle

**DOI:** 10.1186/s43019-019-0014-2

**Published:** 2019-12-18

**Authors:** Jung Ho Noh, Woo Dong Nam, Young Hak Roh

**Affiliations:** 1 Department of Orthopaedic Surgery, Graduate School of Medicine, Kangwon National University, 1 Kangwondaehak-gil, Chuncheon-si, Gangwon-do 24341 South Korea; 2 Department of Orthopaedic Surgery, Ewha Womans University School of Medicine, 1071 Anyangcheon-ro, Yangcheon-gu, Seoul, 07985 South Korea

**Keywords:** Anterior cruciate ligament, Instability, Side-to-side difference, Telos

## Abstract

**Purpose:**

To compare side-to-side difference (SSD) of anterior tibial translation in instrumented stress radiography for each series of anterior cruciate ligament (ACL)-injured subjects according to knee flexion angle.

**Methods:**

Forty subjects who were suspected of having significant ACL injury by manual Lachman test and MRI were recruited for this prospective study. These subjects took stress radiographs for both knees with corresponding knee flexion of 10° (series M1) and 30° (series M2) using Telos stress device. Mean SSDs of M1 and M2 were compared. Sensitivities of M1 and M2 were assessed using the SSD ≥ 3 mm or ≥ 5 mm as a cutoff value.

**Results:**

Mean SSDs in series M1 and M2 were 4.22 ± 3.72 mm and 3.25 ± 3.30 mm, respectively (*p* < 0.001). When 3 mm of SSD was used as a cutoff value, sensitivities of series M1 and M2 were 47.5% (19/40) and 32.5% (13/40), respectively (*p* = 0.171). When 5 mm of SSD was used as a cutoff value, sensitivities of series M1 and M2 were 45.0% (18/40) and 22.5% (9/40), respectively (*p* = 0.033).

**Conclusions:**

Anterior tibial translation on stress radiographs using a Telos device is more prominent when knee flexion angle is 10° compared to that when knee flexion angle is 30°. However, stress radiography using Telos device, either at 10° or 30° of knee flexion, might not be suitable to make decision on surgical treatment due to relatively low sensitivities.

## Introduction

Clinical diagnosis of anterior cruciate ligament (ACL) injury has been well established by abnormal anterior translation of proximal tibia on Lachman stress test and magnetic resonance imaging (MRI) is a most accurate diagnostic tool [1, 2]. Degree of anterior instability on Lachman stress test has been graded to make decisions for surgical treatment or assessment of surgical outcomes and prognosis [3–5]. However, assessment of the anterior tibial translation with manual Lachman stress test might be imprecise, subjective, and non-reproducible due to factors such as clinical experience of physical examiner, muscle relaxation, and inherent knee variability [6, 7]. Several devices for objective assessment of knee instability have been introduced, including KT Arthrometer (MEDmetric Corp, San Diego, CA, USA), Telos stress device (Austin & Associates, Fallston, MD, USA), Rolimeter (Aircast Europa, Neubeuern, Germany), Stryker Knee Laxity Tester (Stryker, Kalamazoo, MI, USA), and Genucom Knee Analysis System (FARO medical Technologies Inc., Montreal, Canada). Stress radiography has been introduced to assess knee instability for long time [8]. Telos stress device is known to be highly reproducible in producing knee stress for imaging [9, 10], and it is easy to interpret its results on simple radiographs compared to other modalities.

Anterior instability of the knee has been known to be more obvious in 10° to 30° of flexion than in 70° to 90° of flexion because the “door stopper effect” of the meniscus can obstruct anterior tibial translation with knee flexion of 70° to 90°. Therefore, Lachman test with knee flexion of 10° to 30° is used more than anterior drawer test with knee flexion of 70 to 90° for ACL-injured knee due to its higher sensitivity [11]. Radiographic assessment with Telos stress device may also be used to assess anterior tibial translation in the same way, and flexion angle of the knee is set to be about 20° for stress radiographs in many researches. However, the actual angle of knee flexion that showed on the figures demonstrating the stress radiographs in published studies does not look consistent, ranging less than 10° to more than 30° [10, 12, 13]. It is important to determine whether patients should be managed surgically or not, to accurately assess clinical outcomes after the management, and to estimate the prognosis. However, based on our experience, anterior tibial translation on stress radiographs is not always consistent as the knee flexion angle varing from 10° to 30°. Additionally, stress radiography sometimes does not give accurate information about the severity of anterior instability of ACL-injured knee despite of obvious findings of complete rupture of ACL on MRI with definite anterior instability with soft endpoint on physical examination. The objective of this study was to compare side-to-side difference (SSD) of anterior tibial translation in stress radiography with knee flexion of 10° and that with knee flexion of 30°.

We hypothesized that SSD was not different with each other according to knee flexion angle.

## Materials and methods

This cohort study was performed at Kangwon National University Hospital between Jun 2016 and Aug 2018 with approval from the Institutional Review Board. Inclusion criteria were: those who showed grade 2+ (5–10 mm of anterior tibial translation) or 3+ (more than 10 mm of anterior tibial translation) with soft endpoint on Lachman test by an experienced knee surgeon and showed significant ACL injury (complete or nearly complete rupture) on MRI. Subjects who showed significant osteoarthritic change on knee radiographs such as osteophyte and marked joint space narrowing, those who had concomitant injuries of other ligaments on the affected knee, those who had history of trauma on the affected or contralateral knee, and those who had concomitant bucket handle tear on MRI which might interfere with anterior tibial translation were excluded from this study. Included subjects underwent arthroscopic examination regardless of anterior tibial translation on stress radiography. Subjects underwent ACL reconstruction when significant ACL injury was verified by arthroscopic examination. Prior to arthroscopic examination and ACL reconstruction surgery, two series of stress radiographs of both knees were taken for each subject. For stress radiography, Telos stress device was used with the knee flexion of 10° (series M1) and 30° (series M2) in lateral decubitus position lying on the corresponding side. Knee flexion angle was defined as the angle formed by anatomical axes (intramedullary center lines) of femur (line connecting the centers of the medullary canal 8 cm and 13 cm proximal to the joint line) and tibia (line connecting the centers of the medullary canal 8 cm and 13 cm distal to the joint line). It was verified by goniometer on radiographs. After three preloading cycles, the heel and thigh were fixed to a rigid rod and the subject was instructed to relax. Stress radiographs were taken with anterior load of 150 N applied to the posterior aspect of proximal tibia. Stress radiographs satisfying all criteria listed in Table 1 were accepted. If the stress radiographs screened by radiologic technician did not meet the criteria, the procedure was repeated,

**Table 1:** Radiographic quality criteria

1. Strict lateral X ray (posterior intercondylar distance < 5 mm)	
2. knee joint flexion angle within ±5°	
3. incident ray at the height of the joint space linking the two tibial plateau	
4. x ray radiographically clear	
5. well adapted piston position (behind the proximal metaphysis of the tibia)	

**Fig. 1:**
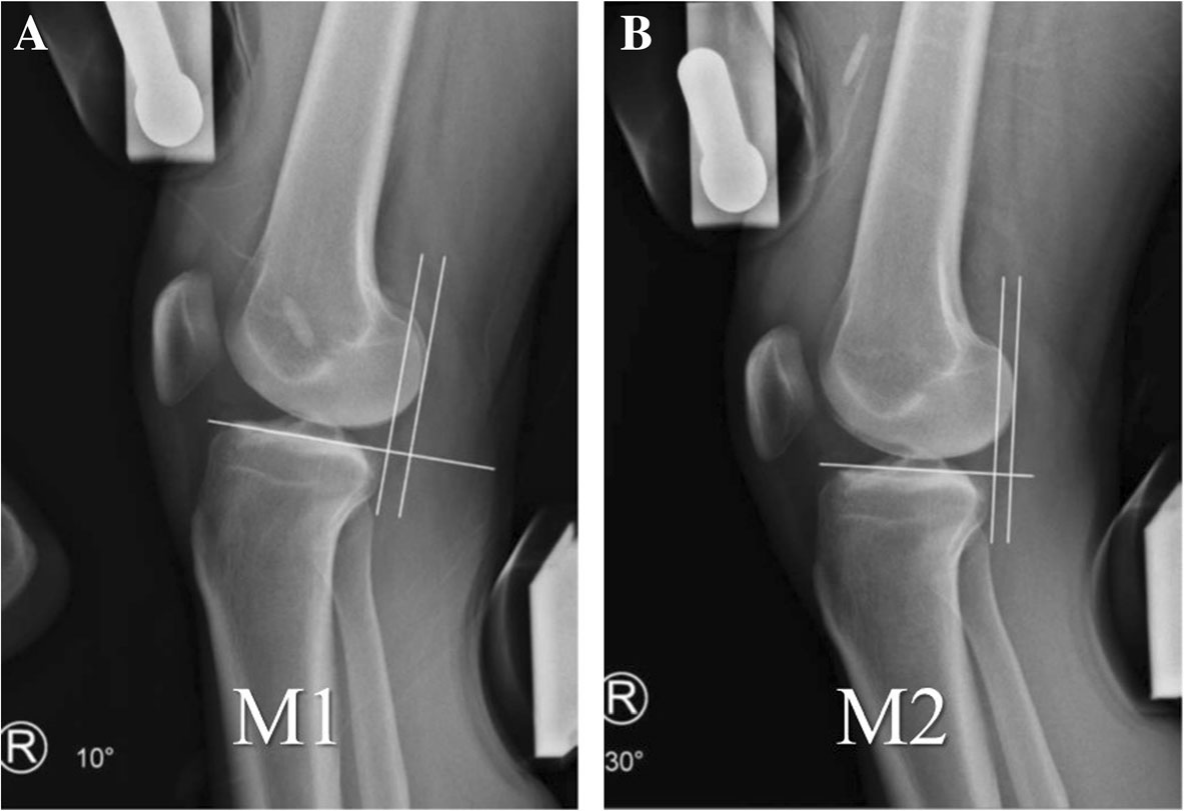
Stress radiography using Telos stress device with knee flexion 10° (**a**) and 30° (**b**). A tangent line to the posterior contour of the medial tibial condyle was drawn perpendicular to the medial tibial plateau. Distance between the line and the posterior aspect of the superimposed posterior femoral condyles was measured

On stress radiographs, a reference line was defined as a tangential line to the medial tibial plateau. From the reference line, two perpendicular lines were drawn. One line was tangential to the most posterior contour of the medial femoral condyle while the other line was tangential to the most posterior contour of the medial tibial condyle. The distance between these two lines was measured on the radiograph of each knee (Fig. 1). The distance between two lines was defined as positive value when the line tangential to tibial condyle was anterior to the line tangential to femoral condyle. Difference between the distances of injured and uninjured knees was defined as SSD for each series of radiographs [14]. The SSD was defined as positive value when the anterior tibial translation was more in the injured knee than that in the uninjured knee.

All radiographic measurements were performed on a Picture Archiving and Communication System (GE Healthcare, Chicago, IL, USA) monitor using a mouse point cursor and an automated computer calculation (precision, 0.01 mm). Measurements were made by two orthopaedic surgeons for each subject to reduce the measurement bias. Measurement values used for statistical analyses were means of measurements by the two observers. Mean values of M1 and M2 were compared. Sensitivities of M1 and M2 were assessed when SSD ≥ 3 mm or ≥ 5 mm was used as cutoff value. The subjects were divided into two groups according to time interval from injury to radiographic assessment (acute phase: ≤3 weeks, subacute or chronic phase: > 3 weeks). Mean values of M1 and M2 were compared and sensitivities of M1 and M2 were assessed for each group.

### Statistical analyses

The sample size was calculated to be 37 subjects for each series with a power of 80% and a significance level of 0.05 to detect difference between M1 and M2 series with SSD of 3.0 mm in a pilot study. Statistical analyses for differences in continuous variables between two groups were performed with unpaired Student *t* test. Differences in categorical variables were analyzed with χ^2^ test. Pearson correlation test was used to determine SSD correlation between series M1 and M2. Inter-observer reliability was assessed by intra-class correlation coefficient (ICC) with 95% confidence intervals (CI). SPSS software (version 19.0; SPSS, Chicago, IL, USA) was used for all statistical analyses. Statistical significance was set at *p* < 0.05.

## Results

Fifty-eight subjects were suspected of having complete or nearly complete rupture of ACL on physical examination and MRI. Of these subjects, eighteen were excluded before arthroscopic examination. Thirteen subjects had concomitant injury of other ligaments. Three subjects had previous trauma on contralateral knee. Two subjects showed marked OA change on the affected knee. One subject refused arthroscopic examination and operation. Therefore, a total of 40 subjects participated in this study. All subjects showed grade 2+ or 3+ anterior instability with soft endpoint on Lachman test. They had complete or nearly complete rupture of ACL on arthroscopic examination. They all underwent ACL reconstruction. Demographics of these included subjects are summarized in Table 2. Inter-observer ICCs for SSDs of series M1 and M2 were 0.979 (95% CI: 0.961–0.989) and 0.982 (95% CI: 0.966–0.991), respectively.

**Table 2:** Demographics of subjects included in this study

Sex (Male:Female)	30:10
Age (years, mean ± SD^*^)	37.8 ± 11.5
Laterality (Right:Left)	21:19
Cause of Injury	
Sports	23
Falldown	8
Traffic accident	5
unknown	4
Time from injury to radiographic assessment (weeks, mean ± SD^*^)	16.6 ± 39.8
Acute phase (≤3 weeks) (Male:Female)	22 (18:4)
Subacute phase (> 3 weeks and < 3 months) (Male:Female)	5 (2:3)
Chronic phase (≥3 months) (Male:Female)	13 (10:3)

^*^standard deviation

**Fig. 2:**
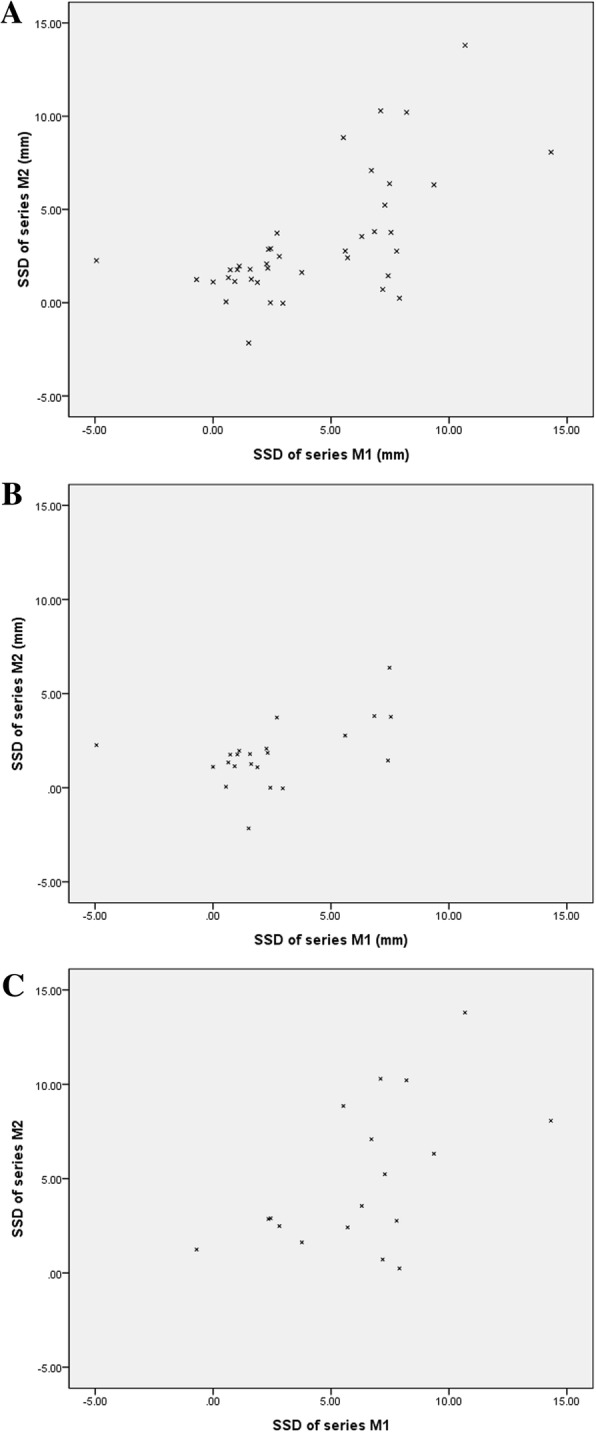
Scattergrams showing the relationship of SSD between series M1 and M2. **a** Total, **b** Subjects with acute phase, and **c** Subjects with subacute or chronic phase

Mean distance between two lines perpendicular to the reference line was 4.38 ± 3.95 mm in injured knee and 0.15 ± 2.04 mm in uninjured knee in series M1, and 3.39 ± 3.55 mm in injured knee and 0.14 ± 2.26 mm in uninjured knee in series M2. Mean SSDs in series M1 and M2 were 4.22 ± 3.72 mm and 3.25 ± 3.30 mm, respectively (*p* < 0.001). The correlation coefficient between SSD of M1 and that of M2 was 0.633 (*p* < 0.001) (Fig. 2a). When 3 mm of SSD was used as cutoff value for surgical treatment, sensitivities of series M1 and M2 were 47.5% (19/40) and 32.5% (13/40), respectively (*p* = 0.171). When 5 mm of SSD was used as cutoff value for surgical treatment, sensitivities of series M1 and M2 were 45.0% (18/40) and 22.5% (9/40), respectively (*p* = 0.033).

In acute phase subjects, mean SSDs in series M1 and M2 were 2.46 ± 2.97 mm and 1.78 ± 1.71 mm, respectively (*p* = 0.001). The correlation coefficient between SSD of M1 and that of M2 was 0.415 (*p* = 0.055) (Fig. 2b). When 3 mm of SSD was set as cutoff value for surgical treatment, sensitivities of series M1 and M2 were 22.7% (5/22) and 22.7% (5/22), respectively (*p* = 1.000). When 5 mm of SSD was used as cutoff value for surgical treatment, sensitivities of series M1 and M2 were 22.7% (5/22) and 4.5% (1/22), respectively (*p* = 0.089).

In subacute or chronic phase subjects, mean SSDs in series M1 and M2 were 6.37 ± 3.45 mm and 5.04 ± 3.90 mm, respectively (*p* < 0.001). The correlation coefficient between SSD of M1 and that of M2 was 0.426 (*p* = 0.078) (Fig. 2c). When 3 mm of SSD was used as cutoff value for surgical treatment, sensitivities of series M1 and M2 were 77.8% (14/18) and 50.0% (9/18), respectively (*p* = 0.083). When 5 mm of SSD was used as cutoff value for surgical treatment, sensitivities of series M1 and M2 were 72.2% (13/18) and 44.4% (8/18), respectively (*p* = 0.091).

## Discussion

The principal finding of this study was that stress radiography showed significantly more sensitivity in determining surgical management for ACL rupture with knee flexion of 10° than that for ACL rupture with knee flexion of 30° when cutoff value of SSD was set to be ≥5 mm. Mean SSD with knee flexion of 10° in stress radiography was greater than that with knee flexion of 30°. However, when subjects were divided into two groups (acute phase and subacute/chronic phase), there was no statistically significant difference in SSD or sensitivity of stress radiography according to knee flexion angle in either of the two groups. This might be due to the small sample size of either group. The distance between two lines perpendicular to the reference line varied widely either in injured or uninjured knee in this study, which means that the anterior instability cannot be assessed with a single knee and should be assessed by comparing the anterior tibial translation between injured and uninjured knees [10, 15].

The advantage of stress radiography is that it is a non-invasive, objective, and reproducible method for recording the amount of anterior translation of skeletal organs, thus eliminating intervention from compliance of soft tissues that might occur with arthrometers. Stress radiography might be used to augment the diagnosis of ACL injury and provide objective grounds for surgical treatment. Several authors have reported the superiority of Telos stress radiography to active stress radiography such as Franklin X-rays for the assessment of anterior tibial translation [9, 16, 17]. However, the accuracy of stress radiography can be affected by other factors such as pain inhibiting muscle relaxation, or inaccurate projection of X-ray.

Precise evaluation of knee instability is important because surgical indication and clinical results are determined by this parameter. SSD of 3 mm or more is generally regarded as clinically significant, and 5 mm of SSD is generally considered as a cutoff value for determining surgical treatment for ACL injury [18]. Many studies evaluating reliability of stress radiography have reported satisfactory results [19–21]. This study assessed the diagnostic value of stress radiography using Telos stress device. The sensitivity of the test was 45.0% for 10° of knee flexion, and 22.5% for 30° of knee flexion with statistically significant difference when a cutoff value was set at 5 mm. When the cutoff value was set at 3 mm, meanwhile, sensitivities were not significantly different between the two series. This fact suggests it might be better to perform stress radiography with knee flexion of 10° in deciding management options for ACL injury. However, this study revealed that sensitivities of the two series were not high enough for diagnosis of clinically significant ACL injury. The sensitivity of stress radiography varies from 67 to 96% in the literature with a cutoff value ranging from 2 to 6 mm. Boyer et al. [22] have reported that the sensitivity of stress radiography is 72% with 5 mm of anterior tibial translation as a cutoff value when a force of 250 N is loaded using Telos device. Beldame et al. [9] have reported a specificity of 59% with 4 mm of anterior tibial translation as a cutoff value when 250 N is loaded using Telos device. These values of sensitivities seem to be low considering that physical examination by an expert has sensitivity of 85% with Lachman test according to a meta-analysis conducted by Benjaminse et al. [23] or the study of Garces et al. [24]. However, these studies did not specify the time interval between injury and the radiologic evaluation.

In current practice, clinical examination and MRI are used as keys to diagnose ACL rupture. However, the stress radiography is used when the clinical examination and MRI results are not consistent, rather than routinely used for diagnostic values. Stress radiography also might be used for prognosis and assessment of postoperative outcomes. It allows surgeon to quantify laxity postoperatively.

Early detection of complete rupture of ACL and adequate surgical treatment are mandatory to restore knee stability because neglecting ACL rupture will make knee instability worse. Results of delayed surgical treatment may be dissatisfying [25–28]. However, muscle guarding due to pain or apprehension can make physical examination or radiographic test for instability less sensitive. Panisset et al. [29] have reported that laxity can increase with time, making it easier to obtain higher sensitivity values for a series of chronic ruptures as opposed to acute ruptures. This study also showed that anterior laxity in subjects with subacute or chronic injury was increased compared to subjects with acute injury.

Most authors have recommended knee flexion angle ranging from 10° to 30° for stress test, especially 20° [9, 13, 30]. However, majority of them did not specify the reference line for knee flexion angle. They have to tolerate errors in knee flexion angle during radiographic examination because it is difficult to achieve and maintain an accurate angle while taking stress radiographs with a stress device. Applying an anterior load to the posterior of the proximal tibia is likely to cause more flexion of the knee than intended. Therefore, it may be important to instruct subjects to keep knee bending at a certain angle. This study revealed that it would be better to keep knee flexion angle at around 10° rather than at around 30°. However, considering the low sensitivity in this study, stress radiography may not be useful for the diagnosis and determination of treatment modalities of ACL injury.

This study has a few limitations. First, this study did not address specificity. It was practically difficult to recruit a normal person to perform stress radiography and MRI. Second, the sample size was too small to analyze the data and assess the sensitivity of stress radiography by dividing subjects to acute and chronic phases. Third, there was no comparison with other testing modalities that could objectively and quantitatively represent anterior tibial translation.

## Conclusions

Anterior tibial translation on stress radiographs using a Telos device is more prominent when knee flexion angle is 10° compared to that when knee flexion angle is 30°. However, stress radiography using Telos device, either at 10° or 30° of knee flexion, might not be suitable to make decision on surgical treatment due to relatively low sensitivities.

## Data Availability

Please contact corresponding author for data request.

## Abbreviations

ACL: Anterior cruciate ligament
CI: Confidence intervals
ICC: Intra-class coefficient
MRI: Magnetic resonance imaging
SSD: Side-to-side difference
